# Characterization of Shikonin Derivative Secretion in *Lithospermum erythrorhizon* Hairy Roots as a Model of Lipid-Soluble Metabolite Secretion from Plants

**DOI:** 10.3389/fpls.2016.01066

**Published:** 2016-07-26

**Authors:** Kanade Tatsumi, Mariko Yano, Kenta Kaminade, Akifumi Sugiyama, Mayuko Sato, Kiminori Toyooka, Takashi Aoyama, Fumihiko Sato, Kazufumi Yazaki

**Affiliations:** ^1^Research Institute for Sustainable Humanosphere, Kyoto UniversityUji, Japan; ^2^Graduate School of Biostudies, Kyoto UniversityKyoto, Japan; ^3^RIKEN CSRSYokohama, Japan; ^4^Research Institute of Chemistry, Kyoto UniversityUji, Japan

**Keywords:** shikonin, *Lithospermum erythrorhizon*, hairy root, lipid secretion, actin filament, brefeldin A body

## Abstract

Shikonin derivatives are specialized lipophilic metabolites, secreted in abundant amounts from the root epidermal cells of *Lithospermum erythrorhizon*. Because they have anti-microbial activities, these compounds, which are derivatives of red naphthoquinone, are thought to serve as a chemical barrier for plant roots. The mechanism by which they are secreted from cells is, however, largely unknown. The shikonin production system in *L. erythrorhizon* is an excellent model for studying the mechanism by which lipophilic compounds are secreted from plant cells, because of the abundant amounts of these compounds produced by *L. erythrorhizon*, the 0 to 100% inducibility of their production, the light-specific inhibition of production, and the visibility of these products as red pigments. To date, many factors regulating shikonin biosynthesis have been identified, but no mechanism that regulates shikonin secretion without inhibiting biosynthesis has been detected. This study showed that inhibitors of membrane traffic strongly inhibit shikonin secretion without inhibiting shikonin production, suggesting that the secretion of shikonin derivatives into the apoplast utilizes pathways common to the ADP-ribosylation factor/guanine nucleotide exchange factor (ARF/GEF) system and actin filament polymerization, at least in part. These findings provide clues about the machinery involved in secreting lipid-soluble metabolites from cells.

## Introduction

Higher plants produce a large number of secondary metabolites, which play various roles in responding to environmental stresses. These natural compounds function as chemical barriers against herbivores, bacteria, and fungi and in protecting plants from harmful UV irradiation (Croteau et al., 2000; Yazaki, 2004; Muranaka and Saito, 2013). Many of these compounds are therefore secreted from plant tissue (Yazaki, 2005).

Most of the metabolites that accumulate in the vacuolar matrix are water-soluble compounds, such as phenol glucosides and alkaloids (Yazaki, 2005; Shitan and Yazaki, 2007). Many transporter molecules responsible for the transport and accumulation of these metabolites been identified, including ATP-binding cassette transporters (Yazaki et al., 2009), multidrug and toxic compound extrusion (MATE)-type transporters (Shitan et al., 2014), and nitrate transporter (NRT) family members (Jørgensen et al., 2015). However, the mechanisms underlying the accumulation of water-insoluble compounds are still largely unknown. Highly hydrophobic metabolites cannot freely move within the cytosol. Moreover, many of these compounds exhibit strong biological toxicity, making it unlikely that they are slowly transported across membranes. Rather, following their biosynthesis, they are likely efficiently excreted from the cytosol into the apoplast. For example, monoterpenes, which are abundant in the leaves of many laminaceous plants, accumulate exclusively in a specialized organ, the glandular trichomes of leaves, and are specifically secreted into the subcuticular cavity, or apoplastic spaces, of glandular trichomes (Lange and Croteau, 1999). Similarly, in citrus species, hydrophobic monoterpenes and furanocoumarins are biosynthesized in epithelial cells of oil glands, with the final products accumulating inside the oil glands, which are apoplastic spaces (Voo et al., 2012).

Taking limited examples of wax and cutin formation at the cell wall, several possible mechanisms for the secretion of these lipophilic compounds are proposed, e.g., oleophilic droplet-mediated mechanism, secretion via plasma membrane-anchoring ER domain where ABC transporters may play a role, cytosolic lipid carrier-mediated mechanism, etc. (Pollard et al., 2008). For lipophilic secondary metabolites, however, secretary processes are largely unknown at the molecular level (Widhalm et al., 2015). Also, biochemical experiments are hardly performed because of the difficulty in collecting appreciable numbers of secretary cells of glandular trichomes or epithelial cells of citrus flavedo.

Hairy root cultures of *Lithospermum erythrorhizon* provide an ideal model to study the mechanisms responsible for the secretion of specialized lipophilic metabolites of plants. Shikonin derivatives are naphthoquinones with C6 side chains that form small fatty acid esters, such as acetyl ester. These compounds play an important role as a chemical barrier against soil-borne microorganisms (Brigham et al., 1999). The core structure of shikonin is biosynthesized from *p*-hydroxybenzoic acid and geranyl diphosphate, which are coupled by a membrane-bound geranyltransferase localized at ER (Yazaki et al., 2002). From this key biosynthetic step, intermediates are to be hydrophobic leading to shikonin formation. Use of shikonin derivatives to study the mechanism of secretion of non-polar specialized metabolites has several advantages. First, shikonin derivatives are water-insoluble metabolites, large amounts of which are secreted from cells and accumulate in apoplastic spaces (Tani et al., 1992, 1993). Second, these natural compounds are red pigments, making their formation easily detectable (Mizukami et al., 1977). Third, the regulation of these root-specific metabolites can be mimicked by hairy root cultures as an experimental model system (Shimomura et al., 1991). This study was therefore designed to analyze the mechanism of secretion of these lipophilic specialized plant metabolites that accumulate in apoplastic spaces.

## Results

**FIGURE 1:**
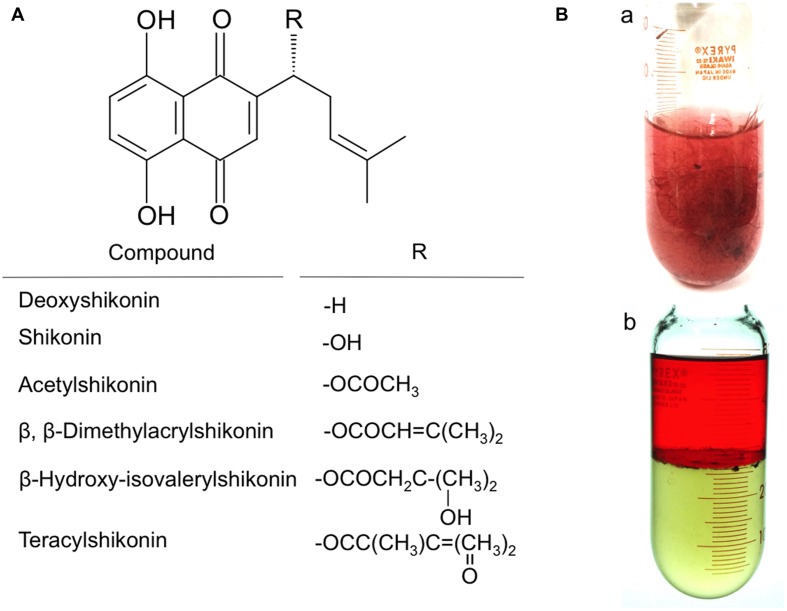
**Hairy root cultures of *L. erythrorhizon* producing shikonin derivatives in M9 medium. (A)** Structures of shikonin derivatives. Red chromophore is naphthoquinone part. Shikonin has a hydroxyl group at the side chain, which is esterified with low molecular weight fatty acids in living cells and free shikonin is almost undetectable from in materials of *L. erythrorhizon*. The major ester is acetyl derivative. **(B)** (a) Glass tube containing a 14-day culture of *L. erythrorhizon* hairy roots in culture medium. Culture medium is turbid as many shikonin derivative-containing granules are suspended (b). Hairy root cultures after partitioning with 1-butanol, revealing that red shikonin derivatives exist outside the root tissues and are lipophilic as being extractable in the organic phase. Root tissues are removed in (b).

Fresh roots of *L. erythrorhizon* contain shikonin esterified with low molecular weight fatty acids, such as acetate, dimethylacrylate, and hydroxyisovalerylate (**Figure 1A**). Hairy roots in liquid cultures of *L. erythrhorizon* were found to have a reddish appearance in M9 medium, due to the excretion of shikonin derivatives into the apoplast (**Figure 1Ba**). Extraction of these root cultures with an organic solvent resulted in the partitioning of the red compounds into the organic phase, indicating the hydrophobicity of shikonin derivatives (**Figure 1Bb**). The excretion of shikonin derivatives and their non-polar nature have been utilized in preparing protein and RNA (Yazaki et al., 2002). Specifically, overlaying of shikonin-producing cultures with liquid paraffin prior to homogenization has been shown to remove these red compounds (Tani et al., 1992, 1993).

**FIGURE 2:**
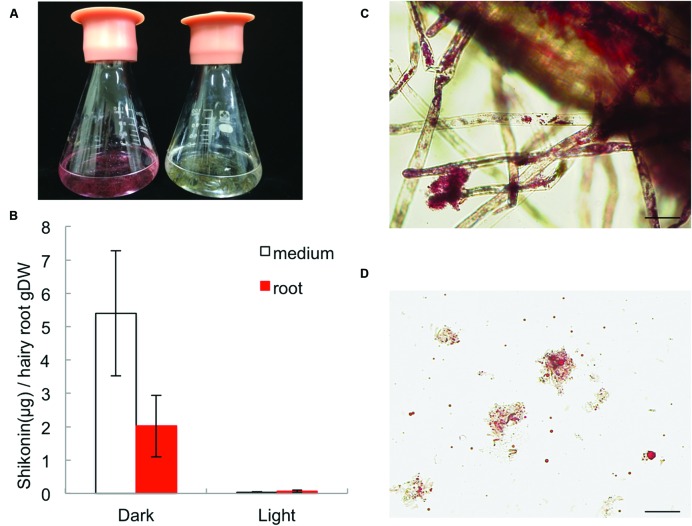
**Production of shikonin derivatives by *L. erythrorhizon* hairy root cultures in M9 medium in the dark and under illumination. (A)** Photographs showing *L. erythrorhizon* hairy roots cultured in M9 medium for 14 days in the dark (left) or under illumination (right). **(B)** Quantitative analysis of shikonin derivatives in triplicate cultures from **(A)**. **(C)** Micrograph of a hairy root in the dark. Shikonin derivatives are present in the red granules attached to the cell surface and root hairs. Bar = 100 μm. **(D)** Filamentous debris with abundant amounts of shikonin-containing red granules suspended in M9 medium. Bar = 100 μm.

In root tissues of *L. erythrorhizon*, shikonin derivatives are mostly accumulated in epidermis and root hairs. Comparing the accumulation rate between root tissues and the cultured medium, shikonin derivatives produced by hairy root cultures were found to root tissues, although higher amounts were also recovered from the culture medium (**Figures 2A,B**). The production of shikonin derivatives was, however, strongly inhibited by irradiation with light. Shikonin derivatives attached to root epidermis appeared as red granules (**Figure 2C**), whereas those detached from the root surface appeared suspended as red granules with filamentous debris in the medium (**Figure 2D**). These findings indicated that hairy root cultures can be used to assess mechanisms regulating shikonin biosynthesis and excretion.

**FIGURE 3:**
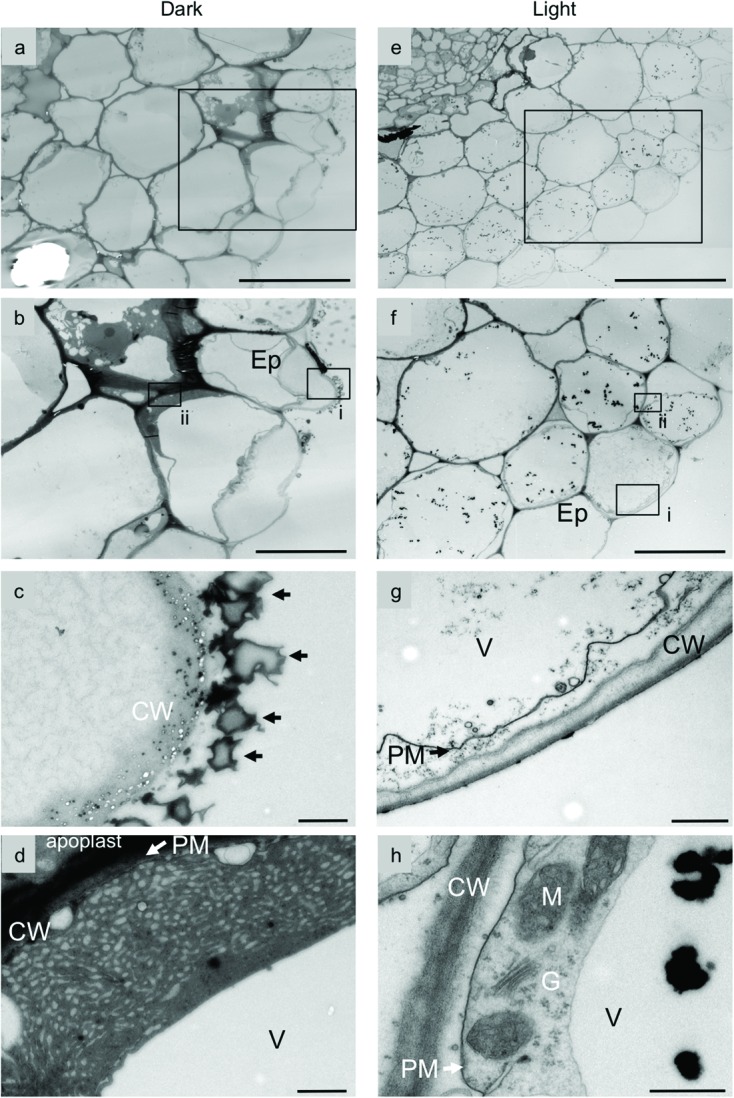
**Transmission electron micrographs of *L. erythrorhizon* hairy roots cultured in M9 medium.** Cross sections of hairy roots cultured in M9 medium in the dark **(a-d)** and under illumination **(e-h)**. Rectangles in **(a,e)** depict the enlargement areas shown in **(b,f)**, respectively. Rectangles (i,ii) in **(b)** depict the enlargement areas shown in **(c,d)**, respectively. Rectangles (i,ii) in **(f)** depict the enlargement areas shown in **(g,h)**, respectively. Arrows in **(c)** are granules containing shikonin derivatives attached on the cell wall. Shikonin derivatives are mostly accumulated in root epidermis and root hairs. Highly developed endoplasmic reticulum (ER) and many small vesicle-like structures are seen in epidermal cells of the hairy roots **(d)**, which are, however, not observed in light grown hairy roots **(h)**. As shikonin derivatives also accumulate between epidermis and cortex, high electron-dense materials in apoplast is presumed as being shikonin derivatives, which are, in contrast, not observed in light grown hairy roots **(h)**. Bars = 50 μm, **(a,e)**; 20 μm, **(b,f)**; 1 μm **(c,d,g)**; 500 nm **(h)**. Ep, epidermal cell; CW, cell wall; G, Golgi body; V, vacuole; M, mitochondria.

The secretion of shikonin derivatives from hairy roots into M9 medium was assessed by transmission electron microscope. **Figure 3a** shows typical root tissues surrounded by epidermal cells; outside the epidermal cell layer, many electron-dense granules were found to be attached to the cell surface outside the cell walls of dark-grown hairy roots (**Figures 3b,c**). These granules appeared identical to the red granules observed by light microscopy, but were not observed in light-grown hairy roots (**Figures 3e–g**). It is noteworthy that many small vesicle-like structures are observed in the cell wall of epidermal cells beneath the shikonin granules attached on the cell surface (**Figure 3c**). The identity of these small vesicles is currently unknown, but appears to be relevant for the shikonin granules on the cell wall surface. Highly developed endoplasmic reticulum (ER) and many small vesicle-like structures were observed in the peripheral regions of dark-grown, shikonin-producing hairy root cells (**Figure 3d**). Frequency of epidermal cells that have highly developed ER-like structures is 24.3%. These characteristic structures were not detected in hairy roots under illumination (**Figure 3h**), suggesting that the secretory pathway involving the ER and small intracellular vesicles is relevant to the secretion of shikonin derivatives. Shikonin pigments also accumulate between epdermis and cortex, high electron-dense materials observed in apoplast of **Figure 3d** is likely to be shikonin derivatives, and the deposition of high electron-dense materials is not clearly seen in hairy root under illumination. These findings are consistent with those observed in dedifferentiated cell cultures, suggesting that dedifferentiated *L. erythrorhizon* cells retain most of the metabolic activity of root epidermal cells (Tsukada and Tabata, 1984).

To date, many chemical and physical factors have been found to regulate the shikonin production, including factors, such as Cu^2+^, acidic oligosaccharides, methyl jasmonate, and cold temperature; and inhibitory factors, such as NH_4_^+^, 2,4-dichlorophenoxyacetic acid (a synthetic auxin), low pH, temperature higher than 28°C and light, especially blue light. Feeding experiments and biochemical and expression analyses of shikonin production in *L. erythrorhizon* showed that all of these inducers and inhibitors acted on biosynthetic enzymes (Lange et al., 1998; Yazaki et al., 1999, 2002; Yamamoto et al., 2000). However, none of these inhibitors affected the secretion of shikonin derivatives without affecting their biosynthesis. We therefore searched for inhibitors of shikonin secretion in hairy root cultures that did not also inhibit shikonin biosynthesis, identifying two compounds, cytochalasin D and brefeldin A (BFA), which are often used in membrane traffic studies.

**FIGURE 4:**
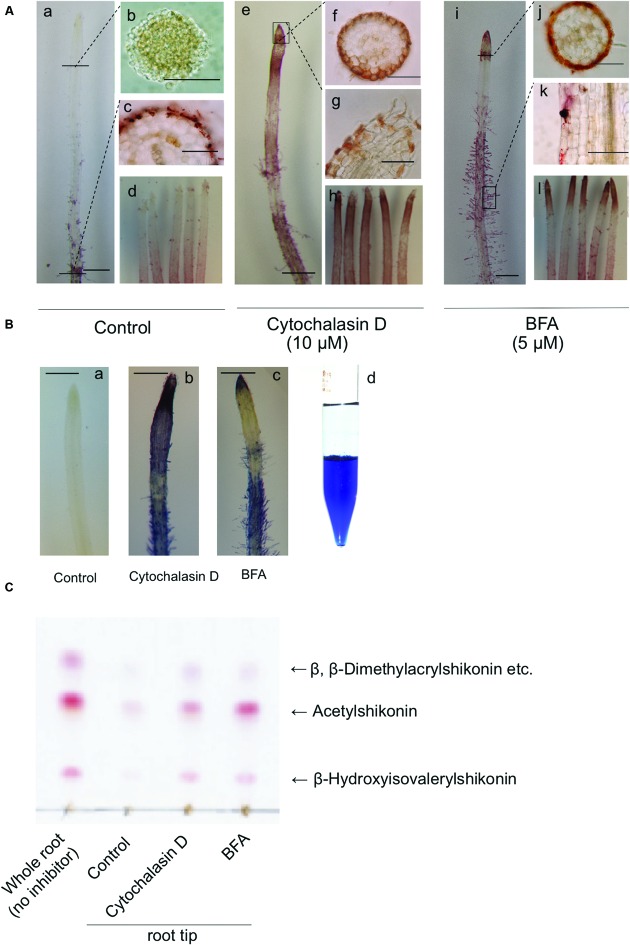
**Accumulation of shikonin derivatives following treatment of root tips with cytochalasin D or BFA in dark-grown hairy roots. (A)** Hairy roots treated with vehicle (20 μL DMSO) (a-d), cytochalasin D (10 μM) (e-h), or BFA (5 μM) (i-l) for 3 days. Bar = 20 μm (a,e,i), 50 μm (b,c,f,g,j,k). **(B)** Treatment of hairy roots with 2.5% KOH spray, which turned shikonin derivatives blue. Partitioning of standard shikonin with diethyl ether and 2.5% KOH, with shikonin present in the aqueous phase as blue pigment **(d)**. Bar = 20 μm (a-c). **(C)** TLC analysis of the acetone extracts of 50 hairy root tips, each treated with vehicle (DMSO), cytochalasin D, or BFA. The solvent system was hexane:acetone:formic acid = 90:10:1.

Accumulation of shikonin derivatives is high in older parts of root tissues, from which root hairs are generated, while being low at root tips (**Figures 4Aa–d**). However, treatment of hairy root cultures with cytochalasin D, an inhibitor of actin filament polymerization, for 3 days resulted in red pigment accumulation from the root cap to the cells in the elongation zone (**Figures 4Ae–g**), with pigmentation observed in almost all root tips (**Figure 4Ah**). Similarly, treatment with BFA, an inhibitor of the adenosine diphosphate (ADP)-ribosylation factor/guanine nucleotide exchange factor (ARF/GEF) protein system, for 3 days resulted in strong pigment accumulation at the root tip region and elongation zone (**Figures 4Ai–l**), with pigmentation observed in almost all root tips (**Figure 4Al**). Cross sectional views of the root tips showed that the red pigment, whether induced by cytochalasin D or BFA, was localized inside the epidermal cells (**Figures 4Af,j**).

Under alkaline conditions, red shikonin derivatives become blue and water-soluble. As the red pigments observed inside the epidermal cells of root tips appeared to more orange than the typical red color of shikonin derivatives observed in hairy roots, it was unclear whether the pigment inside root tips was due to shikonin derivatives. Treatment of these root tips with alkaline spray turned the red pigment, whether induced by cytochalasin D or BFA, to blue in a manner identical to that of pre-existing shikonin derivatives in hairy roots (**Figures 4Bb,c**). In the absence of cytochalasin D or BFA, control hairy roots treated with the alkaline spray showed no evidence of blue color at the root tip (**Figure 4Ba**). Following partitioning with ethyl acetate and 2.5% KOH, standard shikonin was present in the aqueous phase as a blue pigment (**Figure 4Bd**).

To further identify the red pigments, root tips were collected from cytochalasin D- or BFA-treated root tissues and analyzed by thin layer chromatography (TLC; **Figure 4C**). These pigments were found to be shikonin derivatives, made up of the same esters (i.e., β,β-dimethylacryl, acetyl and β-hydroxyisovaleryl shikonin) as control roots.

**FIGURE 5:**
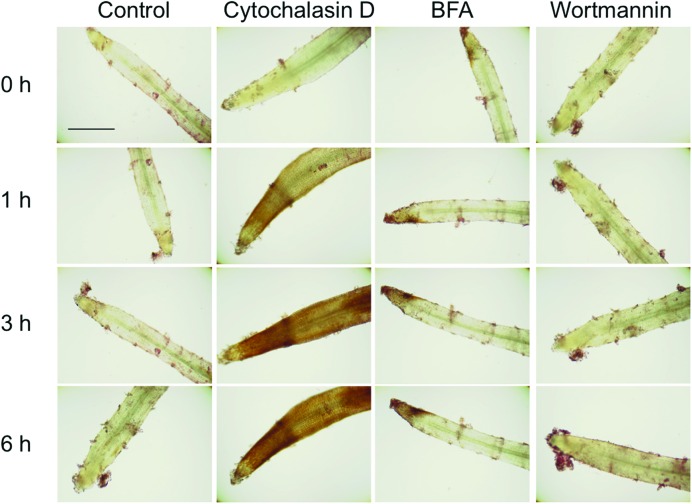
**Accumulation of shikonin derivatives in root tips following treatment with 10 μM cytochalasin D, 10 μM BFA, or 10 μM wortmannin for 1–6 h.** Control is the treatment with DMSO. Bar = 500 μm.

In the above experiments, cultures were treated with cytochalasin D or BFA for 3 days to maximize high accumulation of shikonin derivatives and enable the identification of pigments. To make this experimental system appropriate for biochemical analysis, the treatment time should be as short as possible. Optimization of other conditions showed that treatment with 10 μM cytochalasin D for 1 h or treatment with 10 μM BFA for 3 h resulted in strong shikonin accumulation in both root tips and elongation zones (**Figure 5**). Wortmannin, another inhibitor of membrane trafficking, but differing in mechanism of action from BFA, had no effect on the accumulation of shikonin in tip regions of *L. erythrorhizon* hairy roots. Although some shikonin was observed in border cells after treatment with wortmannin, shikonin was similarly observed in control roots. The identity of these red pigments observed in the short-term treatment was confirmed with KOH treatment, by which shikonin derivatives turn blue (**Supplementary Figure S2**).

**FIGURE 6:**
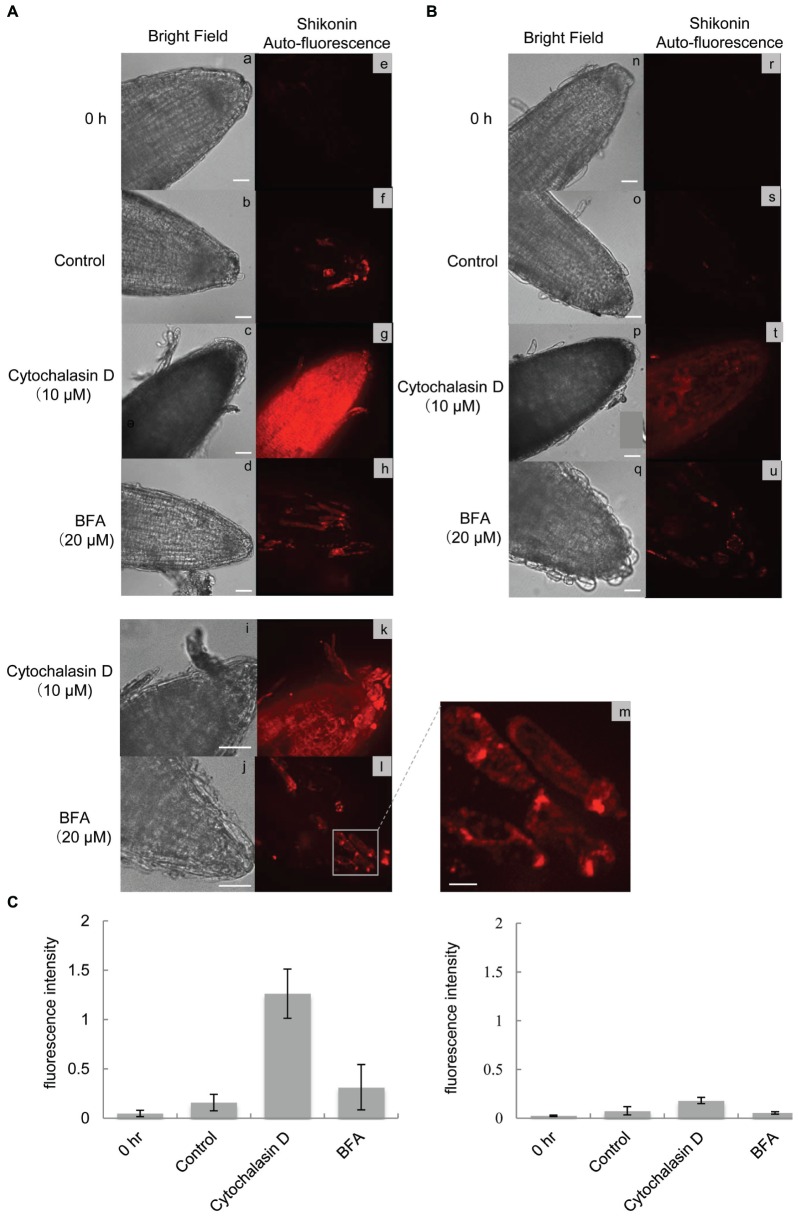
**Confocal microscopic analysis of hairy roots treated with cytochalasin D or BFA. (A)** Fluorescence images of root tips treated with cytochalasin D (10 μM) or BFA (20 μM) in the dark (a-m) for 1 h. The red fluorescence represents the autofluorescence of shikonin derivatives. Bar = 50 μm (a-l), 10 μm (m). **(B)** Fluorescence images of root tips treated with cytochalasin D (10 μM) or BFA (20 μM) under bright light illumination (n-u) for 1 h. Bar = 50 μm. **(C)** Quantification of fluorescence intensity of cells grown in the dark or under bright light illumination for 1 h. Each bar represents the mean ± SD of triplicate determinations.

To further characterize shikonin accumulation patterns in root tip regions following treatment with cytochalasin D or BFA, shikonin derivatives were examined by confocal microscopy. Labeling of shikonin-containing granules and vesicles with fluorescent probes was unsuccessful, because shikonin absorbed their fluorescence. The autofluorescence of shikonin derivatives was therefore directly determined. Measurement of the fluorescence spectrum of shikonin with a fluorophotometer showed maximum emission at 590 nm (**Supplementary Figure S1**). This fluorescence could be detected with a confocal microscope equipped with an EMCCD camera (Andor) and a 656/26 nm emission filter for mCherry. Because this microscope set-up is highly sensitive, low amounts of shikonin derivatives in control root tips could be occasionally detected (**Figures 6Aa–f**). Treatment with cytochalasin D resulted in much higher accumulation of shikonin-derived fluorescence, in a pattern identical to that observed by bright field microscopy (**Figures 6Ac,g**). Hairy roots treated with BFA also showed increased red fluorescence (**Figures 6Ad,h**), primarily inside the cells, with higher magnification showing accumulation in large dotted structure like BFA bodies, which is defined as a specialized region of ER that is observed when vesicle trafficking is prevented by BFA (**Figures 6Aj,l,m**). Cytochalasin D treatment resulted in accumulation of shikonin-derived fluorescence inside the cells (**Figures 6Ai,k**), which revealed small dotted structures in the shorter exposure time (**Supplementary Figure S2**), while their size appeared different from BFA bodies. Red fluorescence was maintained at a low level under illumination, even after treatment with cytochalasin D or BFA (**Figures 6Bn–u**), which was quantified in **Figure 6C**.

## Discussion

The mechanisms underlying the transport and accumulation of secondary metabolites are still fairly uncharacterized. Although transporters responsible for the membrane transport of water-soluble compounds have been identified, less is known about the transport and excretion of lipophilic substances. These compounds are frequently secreted by epithelial cells into oil glands or by secretory cells into the subcuticular cavity. Monoterpenes, for example, are synthesized in plastids, and may be transported outside the cells by hemifusion with the plastidal lumen, a mechanism that may involve as yet unidentified membrane transporters (Wang et al., 2015). This study used a model system to assess the secretion of shikonin derivatives, a group of composite-type lipophilic secondary metabolites containing a shikimate-derived aromatic ring and a mevalonate-derived geranyl moiety and with high affinity for chloroform (Li et al., 1998). Abundant quantities of these lipophilic substances are secreted by root tissues, accumulating in granules attached to the cell surface.

Because of their lipophilicity and biochemical properties, shikonin derivatives that non-specifically stick to proteins, amino acids and inorganic cations are rapidly degraded. Thus, these red pigmented compounds are sequestered in lipid granules present in the phospholipid monolayer and accumulate in apoplastic spaces. To our knowledge, this study is the first to show that this process requires actin and ARF/GEF-related machinery, common to secretory vesicles composed of lipid bilayers, despite large differences between membrane lipid mono- and bi-layers. Oil bodies are lipid monolayers, present in plant cells but not involved in the secretion of lipids, which remain inside these cells as storage organelles for triglycerides, for example. Thus intracellular shikonin vesicles have very different functions than oil bodies. The subcellular accumulation of autofluorescence of shikonin by BFA suggests that a mechanism is involved in the budding of shikonin-containing vesicles from ER in a similar way as vesicle trafficking of protein secretary pathways. The involvement of Golgi apparatus in the shikonin secretion was under discussion, but from our recent observations, Golgi does not appear differently between shikonin-producing and non-producing cells. The inhibition of shikonin secretion by cytochalasin D is indicative that actin filament is necessary for the movement of such oleophilic particles as shikonin-containing vesicles toward plasma membrane. However, the ability of intracellular shikonin vesicles to recognize and fuse to plasma membranes, and the mechanism underlying red shikonin granule formation at the cell surface, remain to be determined.

It is to be noted that highly developed ER with vesicle-like structures is seen in the epidermal cells, where shikonin derivatives are highly accumulated. The frequency was 24.3%, while that of outer cortex is 20.5% and it is much less in inner cortex cells. Shikonin derivatives as final products are observed around the epidermal cells, it might be possible that precursor molecules are supplied from outer cortex cells via vesicle-mediated mechanism. It is also to be clarified how shikonin granules with particular size are formed outside the cell wall. To address this question, an encouraging observation was seen in **Figure 3c**, in which many small vesicle-like structures are seen within the cell wall. As they are much smaller than shikonin granuels these may provide the precursors of shikonin granules that are the final destination of shikonin secretion. Further investigations of those cell wall-embedded small vesicles are necessary in future.

Shikonin derivatives that accumulate in root tip cells following treatment with cytochalasin D or BFA appeared orange in color. The difference in color between shikonin derivatives in root tips and haired regions may be due to a difference in redox state between symplasts and apoplasts. Similarly, ubiquinone is present as a reduced, colorless form within cells, but is air oxidized to a yellow color after isolation.

Under normal conditions, shikonin derivatives mostly accumulate in older parts of root tissues. Root tips also have the potential to produce shikonin, but normally do not (Brigham et al., 1999). This low level of shikonin in untreated root tips may have enabled the effects of BFA and cytochalasin D to be clearly observed. Shikonin induction in cell cultures of *L. erythrorhizon* has been reported to require at least 2-3 days (Yazaki et al., 1997). The benzofuran derivative dihydroechinofuran, a by-product of shikonin production, is sharply induced by transfer to a new medium or treatment with methyl jasmonate (Yazaki et al., 1997). However, dihydroechinofuran is not sequestered in shikonin granules but is secreted from cells in soluble form, even into non-shikonin-producing MS medium, whereas induction of shikonin requires cell maturation for several days (Tani et al., 1993). Our findings suggest that epidermal cells of hairy roots differ from dedifferentiated cultured cells, in that the former have infrastructures necessary for shikonin secretion. Therefore, treatment with cytochalasin D or BFA resulted in shikonin accumulation within a very short period, 1–3 h. This experimental system may enable further biochemical investigations of the mechanism of shikonin secretion.

## Materials and Methods

### Hairy Root Cultures

The hairy root cultures used in this study were established in 1998 (Yazaki et al., 1998) and have since been maintained in M9 medium (Fujita et al., 1981). Assessment by the Japanese Plant Protection Station found no residual *Agrobacterium rhizogenes* in the hairy root cultures.

### Materials

For each analysis, 0.4 g fresh weight of hairy roots were added to 30 mL of M9 medium without auxin and cytokinen and cultured on a rotary shaker (80 rpm) at 25°C for 14 days in the dark or under illumination.

### Quantification of Shikonin Derivatives

*Lithospermum erythrorhizon* hairy roots were cultured in M9 medium with 3 mL liquid paraffin (WAKO) for 3-4 h. After addition of 5 mL hexane, the organic solvent was transferred to a new tube and 2.5% KOH was added. The shikonin was solubilized in 2.5% KOH as a blue pigment. Shikonin concentration in the aqueous phase was determined by measuring its absorbance at 650 nm. Alternatively, hairy roots were harvested by filtration through Miracloth (CALBIO-CHEM. Co.) and dry weight was measured. Shikonin was extracted into chloroform:methanol (2:1 and solubilized in 2.5% KOH as a blue pigment, followed by measurement of absorbance at 650 nm.

### Identification of Shikonin Derivatives

Shikonin derivatives were extracted with methanol from ca. 100 root tips (1.0-1.5 mm) after cytochalasin D or brefeldin A treatment, and the extracts were analyzed by TLC (TLC Silica gel 60, Millipore). The solvent was hexane:acetone:formic acid = 90:10:1.

### Transmission Electron Microscopy (TEM)

Samples were prepared as described (Toyooka et al., 2000) with some modifications. Briefly, hairy roots were fixed in 4% paraformaldehyde, 2% glutaraldehyde, 100 mM sodium cacodylate buffer (pH 7.4) overnight at 4°C, then post-fixed with 1% osmium tetroxide in 50 mM cacodylate buffer for 3 h at room temperature. After dehydration in a graded methanol series (25, 50, 75, 90, and 100%), the samples were embedded in Epon812 resin (TAAB). Ultrathin sections (60-80 nm) were cut by a diamond knife on an ultramicrotome (Leica EM UC7, Leica Microsystems) and placed on formvar-coated copper grids. The ultrathin sections were stained with 4% uranyl acetate followed by lead citrate solution and observed with a JEM-1400 transmission electron microscope (JEOL Ltd.) at 80 kV.

### Vesicle Transport Inhibition Assay

Hairy roots were treated with 10 μM cytochalasin D (diluted from a 2.5 mM stock solution in DMSO, WAKO), 10 or 20 μM BFA (diluted from a 2.5 mM stock solution in DMSO, WAKO), 10 μM wortmannin (diluted from a 2.5 mM stock solution in DMSO, WAKO), or, as control, 4% DMSO. Accumulation of shikonin derivatives was tested by treatment of roots with 2.5% KOH.

### Microscopy

Hairy roots were treated with cytochalasin D or BFA, as described above. The roots were examined with an Axioscope 2 (Zeiss) just after (0 h), and 1, 3, and 6 h after, cytochalasin D or BFA treatment (**Figure 5**) or after 3–5 days (**Figure 4A**). Hairy root sections were prepared by embedding in 5% agarose and cutting into 30-40 μm slices (D.S.K. Microslicer DTK-1000).

### Confocal Microscopy

Hairy roots were treated with cytochalasin D or BFA, as described, and the roots were examined with an Axioplan 2 (Zeiss) microscope equipped with a Yokogawa CSU-X1 spinning disk confocal unit and an Andor ixon+ EMCCD camera just after (0 h) and 1 h after with cytochalasin D or BFA treatment. Shikonin autofluorescense was detected using a 561 nm continuous wave laser (Coherent) and a 656/26 nm emission filter (Semrock). Fluorescence intensity was analyzed by ImageJ software (*n* = 3).

## Author Contributions

The article was written by KY, experimental design was made by KY, FS, TK, TA, KY, instructions were given by AS, FS, KiT, TA, FS, KY, experiments were conducted by KaT, MY, KK, MS.

## Conflict of Interest Statement

The authors declare that the research was conducted in the absence of any commercial or financial relationships that could be construed as a potential conflict of interest.

## Supplementary material

FIGURE S1 **Fluorescence excitation and emission wavelengths of shikonin**.

FIGURE S2 **Root tip of cultured hairy roots treated with cytochalasin D. (A)** Treatment of hairy roots with 10 μM cytochalasin D for 6 h (a), followed by and 2.5% KOH spray (b). Bar = 100 μm. **(B)** Fluorescence images of root tips treated with cytochalasin D (10 μM) for 1 h. Exposure time is half (25 msec) of **Figure 6Ag**. Bar = 100 μm (a,b); 50 μm (c,d).
