# Supplementary figures and images for: Characterization of Shikonin Derivative Secretion in Lithospermum erythrorhizon Hairy Roots as a Model of Lipid-Soluble Metabolite Secretion from Plants
Source: Front Plant Sci. 2016 Jul 26;7:1066. doi: 10.3389/fpls.2016.01066 (PMC4961010; doi:10.3389/fpls.2016.01066)

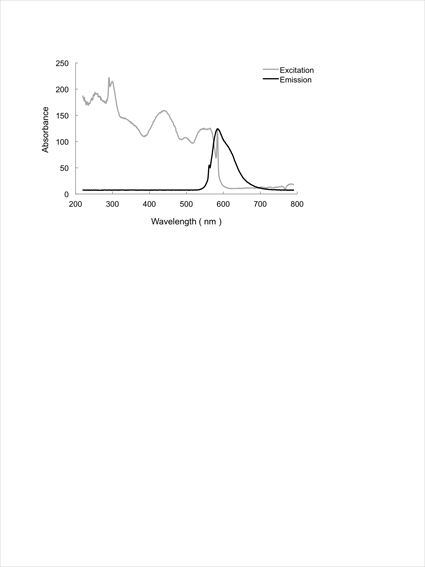

Supplement: FIGURE S1 — Fluorescence excitation and emission wavelengths of shikonin. [file Image_1.TIFF]

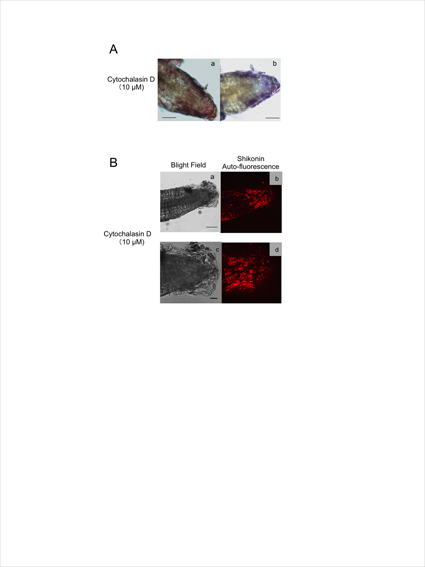

Supplement: FIGURE S2 — Root tip of cultured hairy roots treated with cytochalasin D. (A) Treatment of hairy roots with 10 μM cytochalasin D for 6 h (a), followed by and 2.5% KOH spray (b). Bar = 100 μm. (B) Fluorescence images of root tips treated with cytochalasin D (10 μM) for 1 h. Exposure time is half (25 msec) of Figure 6Ag. Bar = 100 μm (a,b); 50 μm (c,d). [file Image_2.TIFF]
